# Effect of Freeze-Drying on the Antioxidant Compounds and Antioxidant Activity of Selected Tropical Fruits

**DOI:** 10.3390/ijms12074678

**Published:** 2011-07-20

**Authors:** Norshahida Mohamad Shofian, Azizah Abdul Hamid, Azizah Osman, Nazamid Saari, Farooq Anwar, Mohd Sabri Pak Dek, Muhammad Redzuan Hairuddin

**Affiliations:** 1 Department of Food Science, Faculty of Food Science and Technology, Universiti Putra Malaysia, Serdang, Selangor 43400, Malaysia; E-Mails: norshahidams@gmail.com (N.M.S); azosman@putra.upm.edu.my (A.O); nazamid@putra.upm.edu.my (N.S); fqanwar@yahoo.com (F.A.); s_sabrez@yahoo.com (M.S.P.D); chasecult_me@yahoo.com (M.R.H); 2 National Agrobiotechnology Institute, Ministry of Science, Technology and Innovation of Malaysia, Serdang, Selangor 43400, Malaysia; 3 Department of Chemistry & Biochemistry, University of Agriculture, Faisalabad 38040, Pakistan

**Keywords:** drying process, fruits antioxidants, bioactive compounds, ascorbic acid, HPLC, antioxidant attributes

## Abstract

The effects of freeze-drying on antioxidant compounds and antioxidant activity of five tropical fruits, namely starfruit (*Averrhoa carambola* L.), mango (*Mangifera indica* L.), papaya (*Carica papaya* L.), muskmelon (*Cucumis melo* L.), and watermelon *Citruluss lanatus* (Thunb.) were investigated. Significant (*p* < 0.05) differences, for the amounts of total phenolic compounds (TPC), were found between the fresh and freeze-dried fruit samples, except muskmelon. There was no significant (*p* > 0.05) change, however, observed in the ascorbic acid content of the fresh and freeze-dried fruits. Similarly, freeze-drying did not exert any considerable effect on β-carotene concentration of fruits, except for mango and watermelon, where significantly (*p* < 0.05) higher levels were detected in the fresh samples. The results of DPPH (2,2-diphenyl-1-picrylhydrazyl) radical scavenging and reducing power assays revealed that fresh samples of starfruit and mango had relatively higher antioxidant activity. In case of linoleic acid peroxidation inhibition measurement, a significant (*p* < 0.05) but random variation was recorded between the fresh and freeze-dried fruits. Overall, in comparison to β-carotene and ascorbic acid, a good correlation was established between the result of TPC and antioxidant assays, indicating that phenolics might have been the dominant compounds contributing towards the antioxidant activity of the fruits tested.

## 1. Introduction

Consumption of some fruits and vegetable is strongly linked with several health benefits due to their high nutritional value and medicinal properties [1]. Tropical fruits, being rich in functional biaoctives, are valued as one of the potential sources of antioxidants. Antioxidants are believed to control and reduce the oxidative damage in foods and bio-molecules by delaying or inhibiting the oxidation process caused by reactive oxygen species, thus enhancing the shelf-life and quality of the products as well as protecting the biological systems [2]. Antioxidant compounds such as β-carotene, ascorbic acid and phenolics play therapeutic and preventive roles against several diseases such as aging, inflammation and certain cancers [3,4]. Therefore, increased consumption of tropical fruits has been recommended by various health advocates for maintaining good health [5–7].

Post-harvest processes such as drying, cutting, storage, packaging, fermentation, and cooking, *etc*. might affect the phenolics composition and antioxidant activity of foods [8–13]. Freeze-drying is a process whereby water is removed by dehydration, through sublimation of ice in the materials. It is generally recommended for drying of materials containing heat-sensitive antioxidant components such as tocopherols, ascorbic acid, carotenoids and plant phenolics. Freeze-drying is known to extend the shelf-life of foods by preventing the microbial growth and retarding lipid oxidation [14]. It is also applied for long-term storage of foods for the purposes of preserving on industrial scale [8]. Freeze-dried products are believed to have the same characteristics as those of fresh ones. As such, preservation and retention of the attributes such as shape, appearance, taste, nutrients, porosity, color, flavor, texture and biological activity of the fresh samples makes this technique one of the most fascinating and applicable process for drying food materials. Nevertheless, longer drying time is required due to the freeze-dryer’s lower vapor pressure driving force as compared with that of conventional drying methods. Moreover, during freeze-drying treatment, there may be a chance of decline in the content of antioxidants due to degradation of certain compounds. Besides, the freeze-drying operational cost is also high [15].

In some previous studies, losses of food vitamins and nutritional value due to freeze-drying have been reported [16,17]. It is also evident that the composition of some antioxidants and the antioxidant activity of the fruits are affected by freeze-drying [16]. Freeze-drying is used as an alternative to preserve the fruit, nevertheless it might influence the antioxidant properties of the samples processed. The main objective of the present study was to appraise the effect of freeze-drying on the selected antioxidant compounds (total phenolics, ascorbic acid and β-carotene) and antioxidant activity of five commonly consumed tropical fruits.

## 2. Results and Discussion

### 2.1. Moisture Content

The results in Table 1 depict the moisture content of the tested tropical fruits on afresh weight (FW) basis. Watermelon and muskmelon were found to have the highest moisture content (*ca.*92%) whilst papaya had the lowest (80%).

### 2.2. Total Phenolic Compounds (TPC)

The results from the study (Table 2) showed that TPC of fresh and freeze-dried fruits tested varied significantly (*p* < 0.05) ranging from 14 to 181 mg GAE/100 g sample FW. Fresh starfruit (181.71 mg GAE/100 g FW) was found to have the highest TPC, followed by fresh mango (99.69 mg GAE/100 g FW), papaya (67.76 mg GAE/100 g FW), watermelon (29.32 ± mg GAE/100 g FW) and muskmelon (16.71 mg GAE/100 g FW). TPC values reported by other researchers for selected tropical fruits varied from the values obtained in this study, Reporting the following findings:total phenolics content of acidic starfruit (142.9 mg GAE/100 g FW) and sweet starfruit (209.9 mg GAE/100 g FW), mango (56.0 mg GAE/100 g FW) and papaya (57.6 mg GAE/100 g FW) [18]. Meanwhile, a higher TPC was reported in mango (113 mg GAE/100 g FW) but lower in papaya (54 mg GAE/100 g FW) than the present analysis [10]. However, TPC of mango in another study was determined to be 266 mg GAE/100 g FW, significantly higher than our data [19]. There was no available data on TPC of fresh muskmelon (16.71 mg GAE/100 g FW) and watermelon (29.32 mg GAE/100 g FW). Fresh starfruit contained comparable TPC with that of other tropical fruits such as banana (52–231 mg GAE/100 g) and pineapple (47–174 mg GAE/100 g) [19,20], however, the amount was still lower than that reported in blackberry (417–555 mg GAE/100 g FW).

Such differences in the results of TPC compared with other researchers may be linked to different varieties of fruits and the varying antioxidant extraction methods used. The choice of extracting solvents mainly depends on the polarity of the compounds of interest. In the present study, methanol was used, which resulted in higher extraction yields of phenolic compounds due to high polarity. The TPC of methanolic or ethanolic extracts of *Gevuina avellana* seed hulls were considerably higher than that of acetonic extracts [21]. Moreover, factors such as fruit maturity, agroclimate and post harvest storage conditions are known to affect the content of polyphenols in fruits [22–24]. As shown by Table 2, TPC content of fresh starfruit, mango, papaya and watermelon were found to be significantly (*p* < 0.05) higher than that of the freeze-dried samples. The freeze-dried fruits were frozen at −20 °C for 24 h and dried under vacuum condition at −50 °C for 3 days before analysis. It is possible that, through this freezing process, fruit cells might be disrupted, which decompartmentalised certain enzymes, substrates and activators [16,25]. Hence, the increased activity of enzymes upon thawing might have caused degradation of some phenolic compounds [25]. According to another study, onions subjected to freezing showed reduced levels of flavonols, however, those subjected to freeze-drying offered increased amount of flavonols. The increase in flavonol content of freeze-dried onions might be attributed to the liberation of phenolic compounds from the matrix due to freeze-drying [8]. On the other hand, there were no considerable effects observed on the flavonols of freeze-dried onions stored at room temperature during 6 months of storage [8], however a slight increase in flavonols was recorded due to different packaging materials used for storage of fresh-cut sliced onions at 1–2 °C under darkness [11]. As such, the changes in flavonol content during storage of different foods are not clear; the actual amount may increase, decrease or remain unchanged [26]. Such changes are mainly linked to the type of post-harvest processing of foods [27].

### 2.3. Ascorbic Acid Content

Freeze-dried papaya was found to have a significantly (*p* < 0.05) higher ascorbic acid content (16.84 mg/100 g FW) compared to mango (8.34 mg/100 g FW), starfruit (4.67 mg/100 g FW), muskmelon (2.75 mg/100 g FW) and watermelon (2.38 mg/100 g FW). Ascorbic acid content for both fresh and freeze-dried muskmelon and watermelon were not significantly (*p* > 0.05) different from one another, as shown in Table 3. The results obtained from this study were quite different from those reported by other researchers. The ascorbic acid content of fresh starfruit (5.9 mg/100 g FW), mango (19.7 mg/100 g FW), papaya (45.2 mg/100 g FW) and watermelon (3.7 mg/100 g FW) are reported in the literature [28]. Other researchers also reported a higher ascorbic acid content of 74 mg/100 g and 151 mg/100 g in papaya [29,30].

The present experiment reveals that freeze-drying can be used to retain the amount of ascorbic acid as the low temperature processing exerts minimal effect on the deterioration of this water soluble vitamin. Our result is in agreement with that of a previous study which revealed that ascorbic acid content did not vary significantly between the fresh and freeze-dried Sheng-Neu and I-Tien-Hung tomatoes [16]. In another study, freeze-drying was found to retain the maximum amount of vitamin C (ascorbic acid) in papaya [31]. However, it is generally accepted that retention of vitamins also depends on the nature of foods [32].

### 2.4. β-Carotene Content

β-Carotene is ubiquitously present in green leafy and yellow-orange fruits and vegetables. It is very interesting to note that, the highest β-carotene content was found in fresh mango (660.27 μg/100 g FW). This was, however, lower than that found in carrot (6769 μg/100 g) but higher than that of both pumpkin (578 μg/100 g) and tomato (365 μg/100 g) [33]. In a study of six cultivars of Indian mango and two cultivars of papaya, it was found that the content and bio-availability of β-carotene varied among the cultivars tested. Mangoes contained three times more β-carotene than papaya [34]. Similarly, the β-carotene content of Black-gold mango was three times higher than that of papaya [33]. In addition, the β-carotene content of fruits (peach, papaya, apricot and tangerine) may be influenced by the growing conditions, maturity index, post-harvest handling conditions, as well as variety or cultivar [35]. The result of the present study revealed that starfruit had the least β-carotene content compared to the other fruits tested. This is in agreement with the findings of previous studies [23,33] who reported the β-carotene c ontent of s tarfruit to be 20.8 μg/100 g F W a nd 28 μg/100 g FW, respectively. However, the present value was lower than that of starfruit (42 μg/100 g FW) [36]. β-carotene content of fresh mango and watermelon varied significantly (*p* < 0.05) from those of freeze-dried samples while no significant differences were observed for other fruits (Table 4). Carotenoids are present in lipid membranes or stored in plasma vacuoles [35], therefore, it is possible to say that, after degradation of phenolic compounds, second line antioxidants such as β-carotene might have been degraded. It was observed that the concentration of β-carotene in fresh mango and watermelon was reduced by 26% and 43%, respectively as result of freeze-drying.

### 2.5. DPPH (2,2-diphenyl-1-picrylhydrazyl) Radical Scavenging Activity

**Figure 1:**
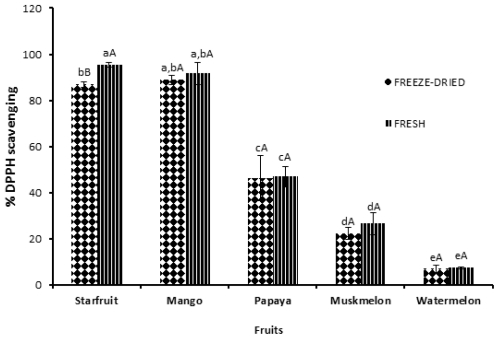
DPPH (2,2-diphenyl-1-picrylhydrazyl) radical scavenging activity of fresh and freeze-dried fruits. Data expressed as % inhibition at 5 mg/mL. Values denoted with the same small letter are not significantly (*p* > 0.05) different among fruits tested. Values with the same capital letter are not significantly (*p* > 0.05) different between the fresh and freeze-dried fruits. Data stand as means of three replicates.

Figure 1 shows the antioxidant activity of the methanolic extract of different tropical fruits as evaluated by free radical scavenging assay. Starfruit and mango extracts (concentration 5 mg/mL) exhibited excellent scavenging effects on DPPH radicals in the range of 87–95%. There were no significant (*p* > 0.05) differences observed for free radical scavenging activity between fresh and freeze-dried fruits, except for fresh starfruit, which showed significantly (*p* < 0.05) higher scavenging activity compared to the freeze-dried sample. Similarly, no significant differences in the scavenging ability of fresh and freeze-dried tomatoes at 4 mg/mL were observed [16]. Starfruit is susceptible to browning during size reduction and processing. Moreover, the freeze-drying process probably enhances the browning reaction. The cut-edge browning is a consequence of enzyme-catalyst browning reactions which involve the oxidation of phenolic compounds by the enzyme activity. The specific enzymes which take part in browning reactions have been generally referred to as polyphenoloxidases (PPO) [37] that act as catalysts in the hydroxylation of monophenols to diphenols and oxidation of diphenols to quinones. The oxidation reaction is relatively rapid compared to hydroxylation and thus contributes more effectively towards the degradation of phenol content as well as antioxidant activity. Different types of fruit have different characteristics of polyphenoloxidase (PPO) [38,39]. There are also differences between PPO from different cultivars and PPO isolated at different stages of fruit maturity. This is based on the reports by various researchers on PPO from peache [40], banana [32], guava [41] and mango [42].

### 2.6. Ferric Reducing Antioxidant Activity

**Figure 2:**
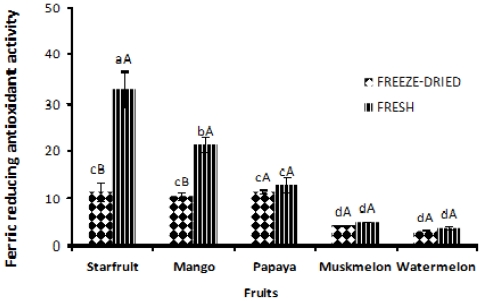
Ferric reducing power of fresh and freeze-dried fruits. Data expressed in μmol/g fresh weight. Values denoted with the same small letter are not significantly (*p* > 0.05) different among fruits tested. Values with the same capital letter are not significantly (*p* > 0.05) different between the fresh and freeze-dried fruits. Data stand as means of three replicates.

Ferric reducing antioxidant power (FRAP) assay measures the total reducing capacity of a compound, based on its ability to reduce Fe^3+^/tripyridyltriazine complex to its blue-colored ferrous form. The results of the present study showed that fresh starfruit exhibited the highest FRAP value of 33.04 μmol TE/g FW (Figure 2). On the other hand, it was noted that fresh and freeze-dried muskmelon as well as watermelon showed the lowest FRAP value revealing no significant (*p* > 0.05) difference between the fruits. Fresh starfruit and mango exhibited significantly (*p* < 0.05) higher FRAP values as compared with that of freeze-dried samples. However, there was no significant (*p* > 0.05) difference in FRAP values between the fresh and freeze-dried papaya, muskmelon and watermelon. A fairly similar trend was seen in the case of FRAP as was reported for that of the DPPH assay. FRAP assay is more suitable for determining the antioxidant activity of water and lipid-soluble components [38].

### 2.7. Antioxidant Activity in Terms of Lipid Peroxidation Inhibition by Conjugated Diene Assay

**Figure 3:**
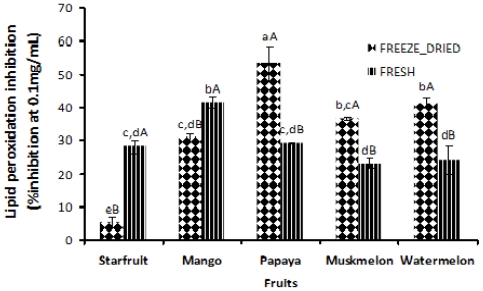
Antioxidant activity (in terms of lipid peroxidation inhibition) as measured by conjugated diene assay of fresh and freeze-dried fruits. Data expressed as % inhibition at 0.1 mg/mL. Values denoted with the same small letter are not significantly (*p* > 0.05) different among fruits tested. Values with the same capital letter are not significantly (*p* > 0.05) different between the fresh and freeze-dried fruits. Data stand as means of three replicates.

The conjugated diene assay measures the ability of antioxidant to inhibit the peroxidation of linoleic acid. In the present study, fresh starfruit (28.2%) and mango (41.5%) exhibited significantly (*p* < 0.05) higher magnitudes of lipid peroxidation inhibition than those of freeze-dried samples (5.28% and 30.92%, respectively) (Figure 3). However, interestingly, freeze-dried papaya (53.3%), muskmelon (36.7%) and watermelon (41.2%) showed significantly (*p* < 0.05) higher antioxidant activity compared to those of fresh samples (29.1%, 23.4% and 24.0%, respectively). As expected, all fruits exhibited a low level of inhibition activity when compared to that of α-tocopherol and BHA (at 0.1 mg/mL), with the inhibition of peroxidation being 87.98% and 98.20%, respectively. The measurement of lipid peroxidation inhibition by employing a conjugated diene assay is useful to assess the antioxidant activity of plant materials. Soybean extracts showed lipid peroxidation inhibition of 55.0% at 1 mg/mL [43] whereas at 5 mg/mL, Taiwan mushroom inhibited peroxidation by 65.2% [44] using conjugated diene assay.

### 2.8. Correlation between Antioxidant Compounds Antioxidant Activity of Fruits

**Figure 4:**
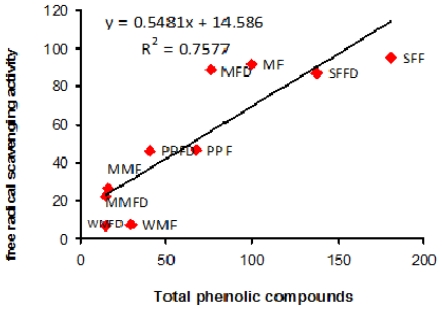
Correlation between free radical scavenging activity and total phenolic compounds of selected fruits.SFFD: starfruit freeze-dried; SFF: starfruit fresh; MFD: mango freeze-dried; MF: mango fresh; PPFD: papaya freeze-dried; PPF: papaya fresh; MMFD: muskmelon freeze-dried; MMF: muskmelon fresh; WMFD: watermelon freeze-dried; WMF: watermelon fresh.

**Figure 5:**
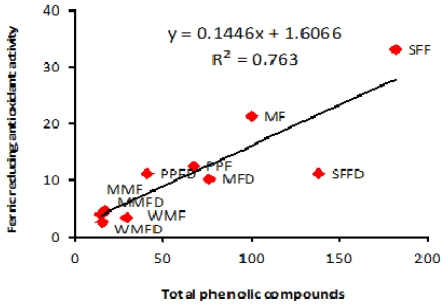
Correlation between ferric reducing antioxidant activity and total phenolic compounds of selected fruits.SFFD: starfruit freeze-dried; SFF: starfruit fresh; MFD: mango freeze-dried; MF: mango fresh; PPFD: papaya freeze-dried; PPF: papaya fresh; MMFD: muskmelon freeze-dried; MMF: muskmelon fresh; WMFD: watermelon freeze-dried; WMF: watermelon fresh.

Coefficient of correlation between the antioxidant compounds and antioxidant activity of the tropical fruits was also studied, as shown in Table 5. Antioxidant activity (free radical scavenging and ferric reducing antioxidant activity) was significantly correlated (*p* < 0.05) with TPC with *r*^2^ = 0.76 and *r*^2^ = 0.76, respectively as shown in Figures 4 and 5. This is not surprising when one considers the similarity between the two assay systems. Significant positive correlation may indicate that the free radical scavenging and ferric reducing antioxidant activities are mainly attributed to the TPC involved. TPC are more likely to be responsible for scavenging most of the free radicals in the fruits studied. Our results are consistent with a previous study which revealed that the content of phenolics in the medicinal and aromatic plant extracts correlates (*r*^2^ = 0.84) significantly with their antiradical activity as measured by a 2,2-azinobis-3-ethyl-benzothiazoline-6-sulfonic acid (ABTS) assay [45]. The phenolic compounds contribute greatly towards antioxidant activity than that of ascorbic acid or carotenoids [6]. Therefore, it could be expected that phenolic compounds might have been the major contributor of antioxidant activity in the presently tested tropical fruits as compared to ascorbic acid or β-carotene.

## 3. Experimental Section

### 3.1. Chemicals

Sodium carbonate, sodium acetate, potassium hydroxide, citric acid, glacial acetic acid, hydrochloric acid, linoleic acid, gallic acid, α-tocopherol, trolox, butylated hydroxyanisole, Folin-Ciocalteau reagent (2N), 2,2-diphenyl-1-picrylhydrazyl (DPPH), 2,4,6-tripyridyl-s-triazine (TPTZ) and ferric (III) chloride hexahydrate were purchased from Sigma (St. Louis, USA). All solvents used, such as methanol, ethanol, hexane, acetonitrile and ethyl acetate were of analytical reagent grade obtained from Fisher Scientific (Leicestershire, UK).

### 3.2. Preparation of Fruit Extracts

Five tropical fruits, namely starfruit (*Averrhoa carambola* L.), mango (*Mangifera indica* L.), papaya (*Carica papaya* L.), muskmelon (*Cucumis melo*), and watermelon (*Citruluss lanatus* Thunb.) were purchased from a local wholesale market in Seri Kembangan, Selangor, Malaysia. All the fruits except for starfruit were washed under tap water and peeled. Starfruit and mango were cut into (2 × 2) cm^2^ whereas papaya, muskmelon and watermelon were cut into (2 × 2 × 2 cm^3^) cubes. Fresh fruits were analyzed immediately. For the freeze-drying experiment, the cubes were packaged in polypropylene plastic containers, and frozen at −20 ± 1 °C for 24 h. The frozen samples were put in the freeze-drier (Labconco, USA) for three days until they were completely dried. Extraction was carried out based on a modified method in the literature [46]. The freeze-dried ground samples were extracted using pure methanol for 1 h at 40 °C using a water bath shaker (Daihan, China). The residues, separated by filtering through Whatman filter paper, were re-extracted twice with the fresh solvent. The three extracts were pooled and then methanol was distilled off at 40 °C using a rotary vacuum evaporator (Büchi, Switzerland). The resulting crude concentrated extracts were used for analysis of total phenolic compounds and antioxidant activity. All analyses were carried out in triplicates.

### 3.3. Determination of Moisture Content

Determination of moisture content was carried out based on the Association of Official Analytical Chemists method [47]. A known amount of fresh fruits were dried in the oven at 105 ± 1 °C. Readings were taken hourly until constant weight was achieved.

### 3.4. Determination of Total Phenolic Compounds (TPC)

Total phenolic compounds were determined using a modified Folin-Ciocalteau colorimetric method as in the literature [48]. Briefly, 0.5 mL of diluted extract was mixed with 0.5 mL of Folin Ciocalteu’s reagent. After 3 min, 10 mL of saturated 7.0% Na_2_CO_3_ was added to the mixture and it was incubated in the dark for 1 h. The absorbance of blue-colored complex solution was then read at 725 nm using UV-1650 PC spectrophotometer (Shimadzu, Japan). Different concentrations of gallic acid (0.02–0.10 mg/mL) were used to construct a calibration curve. The results were expressed as miligram of gallic acid equivalent per hundred gram of fresh weight (mg GAE/100 g FW).

### 3.5. Determination of Ascorbic Acid

Ascorbic acid content was determined using High Performance Liquid Chromatography (HPLC) following a modified method in the literature [49]. Five grams of fruit sample was extracted with 200 mL of 3% w/v citric acid. The mixture was then centrifuged at 10000 rpm (14784 g) for 5 min at 25 °C using Avanti centrifuge J-25 (Beckman Coulter, USA) machine. The supernatant was then filtered using C_18_ Sep-pak cartridge and 0.45 μm membrane filter, prior to HPLC injection. The HPLC system consisted of a Waters 460 pump, UV-VIS detector (Waters, USA) and run by Empower Pro software. The mobile phase used in isocratic elution was acetonitrile-methanol (88:20 v/v) at a flow rate of 1 mL/min. A reversed phase column was used, μBondapak C_18_ column (300 nm, × 3.9 mm, 125 Ǻ, 10 μm). Detection was performed at 234 nm. Results obtained were expressed as miligram per hundred gram fresh weight (mg/100 g FW).

### 3.6. Determination of β-Carotene

β-carotene quantification was made based on the method in literature [33] with some modifications. Five grams of fruit sample was saponified with 20 mL of 95% ethanol and 5 mL of 100% Kalium hydroxide (KOH) and refluxed for 30 min at 85 °C. The mixture was extracted with hexane until the samples became colorless. The extracted sample was then filtered through a 0.45 μm nylon membrane filter and analyzed using reversed-phase high performance liquid chromatography (RP-HPLC) system consisting of a Waters 460 pump, UV-VIS detector (Waters, USA) and run by Empower Pro software. The test solution was injected under isocratic conditions into the μBondapak C_18_ column (300 nm, × 3.9 mm, 125 Ǻ, 10 μm) with a ternary mixture of acetonitrile-methanol-ethyl acetate (88:10:2 v/v) as mobile phase with the flow rate of 1.0 mL/minute. Detection was performed at 436 nm. Results obtained were expressed as microgram per hundred gram fresh weight (μg/100 g FW).

### 3.7. Determination of DPPH Free Radical Scavenging Activity

Free radical scavenging activity of the fruit extracts was carried out according to the modified method in the literature [50]. Briefly, 3.5 mL of methanolic solution (25μg/mL DPPH) was added to 0.5 mL extract at different concentrations. The reaction mixture was then vortexed and kept at room temperature for 30 min. The absorbance was then measured at 515 nm using UV-1650 PC spectrophotometer (Shimadzu, Japan). The results were expressed in percent inhibition at 5 mg/mL. The antioxidant activity was calculated as:

$$\text{AOA }(\%)=[(\Delta {\text{A}}_{515\text{nm}}\;\text{of control}-\Delta {\text{A}}_{515\text{nm}}\;\text{of sample})/\Delta {\text{A}}_{515\text{nm}}\;\text{of control}]\times 100\%$$

### 3.8. Determination of Ferric-Reducing Antioxidant Activity

Ferric-reducing antioxidant power (FRAP) assay was carried out according to the method in the literature [51] with slight modifications. An aliquot of 3.0 mL of FRAP reagent (25 mL of 300 mM acetate buffer (3.1 g C_2_H_3_NaO_2_·3H_2_O and 16 mL C_2_H_4_O_2_, pH 3.6), 2.5 mL of 20 mM FeCl_3_·6H_2_O solution and 2.5 mL 10 mM TPTZ (2,4,6-tripyridyl-s-triazine solution in 40 mM HCl) was added to a test tube containing 200 μL of extract. The mixture was allowed to stand at 37 °C in the darkness; the absorbance was measured at 593 nm after 30 min. The final results were expressed as micromole Trolox Equivalent per gram fresh weight (μmol TE/g FW).

### 3.9. Determination of Antioxidant Activity by Inhibition of Linoleic Acid Peroxidation

The antioxidant activity of fresh and freeze-dried fruit’s extracts was also determined using conjugated diene method [52]. Briefly, 2 mL of 10 mM linoleic acid emulsion at pH 6.5 was mixed with each fruit extract (1–20 mg/mL) and dissolved in 100 μL methanol in a test tube. The mixture was kept in the dark for 15 h at 37 °C. The incubated mixture was then added to 6 mL of 60% methanol. The absorbance was measured at 234 nm. A value of 100% inhibition indicates the strongest antioxidant activity in the sample. The results were expressed in percent inhibition at 0.1 mg/mL. α-Tocopherol and butylated hydroxyanisole (BHA) were used as positive controls. The antioxidant activity was calculated as:

$$\%\;\text{Inhibition of peroxidation}=[(\Delta {\text{A}}_{234\text{nm}}\;\text{of control}-\Delta {\text{A}}_{234\text{nm}}\;\text{of sample})/\Delta {\text{A}}_{234\text{nm}}\;\text{of control}]\times 100$$

### 3.10. Statistical Analysis

Three different samples of each of the five tropical fruits were assayed. Measurements were carried out in triplicates and data obtained from experiments were gathered and analyzed using the Statistical Package for the Social Sciences (Version 16.0). Analysis of Variance was used to determine significant difference between fresh and freeze-dried fruits for antioxidant compounds and activity. Significant difference was determined at *p <* 0.05.

## 4. Conclusions

The results of the present study reveal that freeze-drying can be explored as a viable method for processing tropical fruits retaining the maximum amount of their naturally occurring ascorbic acid. However, this technique can noticeably affect the composition of some other antioxidant components and antioxidant activity of the fruits. Besides this, the results of the study showed that both fresh starfruit and mango are good sources of antioxidants, compared to the other fruits that were tested. Further research on the structural elucidation of the tropical fruits’ individual phenolic compounds and evaluation of their mechanisms of action and biological principles using some *in-vivo* models is recommended.

## Figures and Tables

**Table 1:** Common and scientific names and moisture content of tropical fruits.

Common Name	Scientific Name	a Moisture (%)
Starfruit-B10	*Averrhoa carambola* L.	91.25 ± 0.14
Mango-Chokanan	*Mangifera indica* L.	88.67 ± 0.44
Papaya-Foot Long	*Carica papaya* L.	79.75 ± 0.18
Muskmelon-Sunmelon	*Cucumis melo* L.	92.38 ± 0.17
Watermelon-redmelon	*Citruluss lanatus* (Thunb.)	92.47 ± 0.12

a Data given are mean ± standard deviation for three different samples of each fruit, analyzed individually in triplicate (*n* = 3 × 3).

**Table 2:** Total phenolic compounds of fresh and freeze-dried fruits.

Fruits	Fresh a	Freeze-Dried a
Starfruit	181.71 ± 8.83 ^bB^	137.95 ± 4.31 ^cC^
Mango	99.69 ± 8.70 ^dB^	76.57 ± 8.11 ^eC^
Papaya	67.76 ± 7.36 ^eB^	40.84 ± 6.74 ^fC^
Muskmelon	16.71 ± 1.40 ^gB^	14.97± 1.36 ^gB^
Watermelon	29.32 ± 1.06 ^f,gB^	15.18 ± 2.95 ^gC^

a Data expressed in mg GAE/100 g are fresh weight basis. Values denoted with the same small letter within the same column are not significantly (*p* > 0.05) different among fruits tested. Values with the same capital letter within the same row are not significantly (*p* > 0.05) different between the fresh and freeze-dried fruits. Data stand as means of three replicates.

**Table 3:** Ascorbic acid content of fresh and freeze-dried fruits.

Fruits	Fresh a	Freeze-Dried a
Starfruit	4.99 ± 0.63 ^dB^	4.67 ± 0.42 ^dB^
Mango	8.36 ± 2.33 ^cB^	8.34 ± 1.74 ^cB^
Papaya	16.57 ± 0.36 ^bB^	16.84 ± 2.31 ^bB^
Muskmelon	2.24 ± 0.35 ^eB^	2.75 ± 0.16 ^eB^
Watermelon	1.75 ± 0.37 ^eB^	2.38 ± 0.11 ^eB^

a Data expressed in mg/100 g are fresh weight basis. Values denoted with the same small letter within the same column are not significantly (*p* > 0.05) different among fruits tested. Values with the same capital letter within the same row are not significantly (*p* > 0.05) different between the fresh and freeze-dried fruits. Data stand as means of three replicates.

**Table 4:** β-carotene content of fresh and freeze-dried fruits.

Fruits	Fresh a	Freeze-Dried a
Starfruit	30.79 ± 3.37 ^gB^	25.94 ± 2.15 ^gB^
Mango	660.27 ± 61.06 ^bB^	487.34 ± 29.72 ^cC^
Papaya	243.26 ± 28.55 ^eB^	223.42 ± 24.08 ^eB^
Muskmelon	508.18 ± 13.72 ^cB^	523.26 ± 2.43 ^cB^
Watermelon	290.37 ± 16.96 ^dB^	165.21 ± 5.89 ^fC^

a Data expressed in μg/100 g are fresh weight basis. Values denoted with the same small letter within the same column are not significantly (*p* > 0.05) different among fruits tested. Values with the same capital letter within the same row are not significantly (*p* > 0.05) different between the fresh and freeze-dried fruits. Data stand as means of three replicates.

**Table 5:** Correlation (correlation coefficient, *r*^2^ data) between the antioxidant compounds and the antioxidant activity of selected fruits.

	TPC	AA	BC
DPPH	0.758 *	0.053	0.062
FRAP	0.763 *	0.005	0.077
CD	0.148	0.105	0.178

TPC: total phenolic compounds; AA: ascorbic acid; BC: β-carotene; DPPH: (2,2-diphenyl-1-picrylhydrazyl); FRAP: ferric reducing antioxidant power; CD: conjugated diene.

* Correlation is significant at 0.05 level (1-tailed).
